# Supplementary material for: Variation analysis of growth traits and medicinal components in different provenances of Polygonatum cyrtonema based on heterogeneous garden experiment
Source: PLoS One. 2026 Apr 10;21(4):e0346920. doi: 10.1371/journal.pone.0346920 (PMC13068254; doi:10.1371/journal.pone.0346920)
Supplement: Attachment — Submitted filename: Response to reviewers-Polygonatum cyrtonema - resubmission.docx [file pone.0346920.s003.docx]

Dear editor.

Thank you for your kind letter about my manuscript “Variation analysis of growth traits and medicinal components in different provenances of *Polygonatum cyrtonema* based on heterogeneous garden experiment” (PONE-D-25-60346) on Nov. 14. 2025. We would like to express our great appreciation to you and the reviewers for patient work on our paper. We have finished revising the manuscript and would like to resubmit it again.

We revised the manuscript in accordance with the reviewers’ comments, and carefully proof-read the manuscript. Here below is our description on revision according to the reviewers’ comments.

**Reviewer #1**

Comments: *Reduce the length of the abstract.*

Response: We significantly reduced the length of the abstract, from 371 words to 248 words **in the new lines 13-37**.

Comments: *Streamline the introduction and make it more focused on the aims of the study.*

Response: We have rewritten the introduction section to make it more concise and highlight the research purpose **in the new lines 41-94**. The words had been reduced from 695 to 573 words.

Comments: *If possible, seek proofreading assistance from a native English speaker (e.g., line 62: “current research”?).*

Response: We revised the “Current research” into “Pervious research” (in the new line 57) and polished the manuscript.

Comments: *Justify why only 10 random plants were analyzed out of the 30 plants obtained from 300 rhizomes collected from a single provenance.*

Response: We revised the relevant descriptions in the method. In fact, for each provenance, 90 samples were collected for the experiment. There were 3 experimental plots in each location, and 10 samples in each plot. **It has been added in new lines 111-128.**

Comments: *Provide at least a brief description of the methodology used for estimating polysaccharides, saponins, total phenolic, and flavonoid contents.*

Response: We briefly described the methods for measuring polysaccharides, saponins, total phenolic, and flavonoid contents. **It has been added in new line 130-162:**

The total polysaccharide content was determined using the phenol-sulfuric acid method: approximately 0.05 g of dried sample was weighed, extracted with hot water, and subjected to alcohol precipitation. The supernatant was reacted with phenol-concentrated sulfuric acid reagent at 90°C for 20 minutes, and the absorbance was measured at 490 nm. Glucose was used as the standard for quantification. The total phenol content was determined by the Folin-Ciocalteu method: approximately 0.02 g of dried sample was extracted with 60% ethanol and then reacted with tungstic acid reagent at 25°C for 10 minutes. The absorbance was measured at 760 nm, and gallic acid was used as the standard for calculation. The flavonoid content was measured using the aluminum nitrate colorimetric method: the sample extract was reacted with alkaline aluminum nitrate reagent at 25°C for 15 minutes, and the absorbance was measured at 510 nm. Rutin was used as the standard for calculation. The total saponin content was determined by the vanillin-perchloric acid method: after ultrasonic extraction and evaporation to dryness, the sample was reacted with the coloring reagent at 55°C for 20 minutes, and the absorbance was measured at 589 nm. Oleanolic acid was used as the standard for calculation. All contents were expressed per gram of dry weight (mg·g^-1^).

Comments: *Improve the clarity of Figure 2.*

Response: We have integrated Figures 2, 3, and 4 from the original manuscript into a new Figure 2, and improved its clarity.

Comments: *Most importantly, use the same samples for more advanced analyses, such as GC–MS or HPLC, to determine the exact compounds present in the tested chemotypes.*

Response: In our future research, we will utilize GC-MS or HPLC for detailed analysis of exact compounds.

**Reviewer #2**

**Introduction and Objectives**

Comments: *The introduction is comprehensive but quite dense. Consider summarizing the main research gaps more succinctly.*

Response: We have rewritten the introduction section to make it more concise and highlight the research purpose in the new line 41-94. The words had been reduced from 695 to 573 words.

Comments: *Please clarify the rationale for the choice of only three trial sites within Zhejiang Province—is this sufficient to represent the species' ecological range?*

Response: In the introduction, we have added the reasons for choosing these three locations, which represent the three typical topographical features of East China: Longyou (low mountainous basin), Lin'an (mid-high mountainous area), and Jinyun (typical mountainous region).

Comments: *Are the 11 provenances representative of the species' entire distribution in China, or biased toward Eastern China?*

Response: The 11 provenances biased toward Eastern China.

Comments: *Provide more details on the commercial assay kits used for quantifying medicinal components: kit names, manufacturers, protocol references, and sensitivity/accuracy.*

Response: The polysaccharides, saponins, total phenols and flavonoids, in P. cyrtonema rhizome were determined using kits (Suzhou Comin Biotechnology Co., Ltd., Suzhou, China) in accordance with the kits’ instructions. It has been added in new line 141-145.

Comments: *Clarify if the rhizomes from each provenance were collected from multiple wild plants to ensure genetic heterogeneity.*

Response: We thank the reviewer for raising this critical point regarding genetic heterogeneity, which is essential for the validity of our provenance trial. We can confirm that a specific sampling strategy was employed to ensure the genetic heterogeneity of each provenance.

The detailed procedure was as follows:

Multi-Point Sampling Strategy: For each geographic provenance, rhizomes were not collected from a single plant or location. Instead, we selected 3-5 distinct wild sub-populations within the provenance's natural distribution area, with each sub-population spaced at least 100 meters apart.

Multiple Plants per Sub-population: From each of these sub-populations, we randomly collected rhizomes from multiple (at least 5) healthy, mature wild plants.

Bulk Provenance Sample: The rhizomes from all sub-populations within a single provenance were then thoroughly mixed to create a single, bulk sample that is genetically representative of that provenance for this multi-site trial.

This “multi-point, bulk sampling” approach was designed specifically to capture the intrinsic genetic variation within each provenance. Therefore, the differences observed among provenances in our study robustly represent divergence at the population level and are not attributable to the genetic makeup of a few individual plants.

Comments: *Were any soil or microbiome characteristics of the three experimental sites measured or considered during analysis?*

Response: Firstly, in our study, the primary objective of establishing these three heterogeneous garden sites was precisely to capture a range of uncontrolled, real-world environmental variation (including inherent differences in soil, microbiome, and microclimate) to rigorously test for Genotype-by-Environment (G×E) interactions. The highly significant "Site" effect and "Provenance × Site" interaction we reported for both growth and medicinal traits confirm that these locations provided meaningfully different environmental pressures, even without quantifying every individual soil and microbiome parameter.

Secondly, we acknowledge that a deeper analysis of the soil microbiome would be a valuable avenue for future research to mechanistically explain the specific microbial drivers behind the observed G×E interactions. **It has been added in new line 478-488.**

Comments: *Explain the statistical approach chosen for PCA and clustering, particularly the choice of variables included, were all traits standardized?*

Response: Yes, all the data have been standardized. “Prior to PCA and cluster analysis, the raw data were standardized using the formula Zij=(Xij−μi)/σi, where Zij is the standardized value of the “i”-th trait for the “j”-th provenance, Xij is the original measured value, μi is the mean of the “i”-th trait, and σi is the standard deviation of the “i”-th trait.” **It has been added in new line 168-171.**

**Results**

Comments: *In Table 3 and 4, several values have high coefficients of variation (CV). Can the authors comment on the biological significance and whether these variations might impact practical breeding?*

Response: We conducted a reanalysis of the data, adding phenotype coefficient of variation, genetic coefficient of variation, and provenance repeatability indicators **(in the new lines 227-238)**, and discussed and explained the excessively high indicators **(in the new lines 326-363)**.

Comments: *The positive correlation between saponin content and altitude/latitude is interesting. Can authors hypothesize on potential physiological mechanisms?*

Response: We discussed this interesting mechanism in the newly revised manuscript:

Higher altitude, warmer climate, and lower latitude and longitude were found to favor saponin accumulation, consistent with studies on *Paris polyphylla* [48] and *Bupleurum chinense* [49]. The Jinyun experimental site in this study showed significantly higher saponin content than other sites. Located in the western mountainous area of Zhejiang Province with complex terrain, significant temperature variations, and uneven seasonal precipitation distribution, Jinyun’s environmental conditions support the hypothesis that such stress factors stimulate the synthesis of defensive secondary metabolites like saponins [50]. Our results strongly support this theory, indicating that saponins may serve as key chemical defense compounds in *P. cyrtonema*’s adaptation to specific alpine environmental stresses. **It has been added in new line 410-419.**

Comments: *The negative relation of flavonoids with precipitation reported seems contradictory to some literature. Can authors provide further discussion or references?*

Response: We also discussed this in the newly revised manuscript:

The significant negative correlation between flavonoid accumulation and annual rainfall in P. cyrtonema resembles patterns observed in *Dendrobium officinale* [51], suggesting that relatively arid conditions may promote flavonoid synthesis. The underlying mechanism involves drought stress typically leading to reactive oxygen species (ROS) accumulation in plants. As important antioxidants, flavonoids are upregulated through activated synthesis pathways to scavenge ROS and protect cellular structures. **It has been added in new line 420-426.**

**Discussion**

Comments: *The discussion on environmental regulation of traits is well-developed. Consider providing a more detailed comparison with similar medicinal plants.*

Response: In our discussion of the geographic–climate correlation, we compared our focal species, Polygonatum cyrtonema, with that of four other medicinal plants—*Dendrobium officinale*, *Xanthoceras sorbifolium*, *Paris polyphylla*, and *Bupleurum chinense*.

Comments: *The authors mention limitations related to site number and environmental factors. Could they suggest directions for future studies, such as controlled environmental manipulations?*

Response: During our discussion, we proposed some future research directions: Future work should expand to representative ecoregions (e.g., southwestern mountains, North China Plain), employing integrated transcriptomic-metabolomic analyses to decipher "gene-environment-metabolite" regulatory networks. Longitudinal tracking (≥5 years) of provenance stability under climate change scenarios is also essential to assess adaptive evolution and establish a robust scientific framework for sustainable resource utilization. **It has been added in new line 482-488.**

Comments: *The practical recommendations for cultivating saponin-rich and flavonoid-rich provenances are valuable. Could the authors comment on potential trade-offs or challenges in implementing these?*

Response: During our discussion, we analyzed and provided suggestions on the above issues: In summary, the morphogenesis and medicinal component accumulation in *P. cyrtonema* are driven by environmental factors across different dimensions: growth traits primarily respond to hydrothermal conditions (temperature and rainfall), while key medicinal components are more associated with stress factors (altitude, drought). This finding has significant practical implications for breeding programs. For high saponin content, provenances from high-altitude, warm, low-sunshine regions should be prioritized; for high flavonoid content, provenances from relatively arid regions may be preferable. Notably, both plant height and saponin content showed positive responses to annual temperature, suggesting the potential for synergistic enhancement of growth and saponin accumulation in warm regions, providing valuable screening clues for developing high-yield, high-quality varieties. **It has been added in new line 427-437.**

**Formatting and Presentation**

Comments: *Some figures (like correlation matrices) could benefit from clearer legends or color coding to aid interpretation.*

Response: We have recreated the correlation analysis chart to assist readers in better understanding the content. Please refer to the new Figure 2 for details.

**Reviewer #3**

Comments: *Line 133: “eleven wild P. cyrtonema provenances were collected from four provinces” very confusing as what authors wanted to convey here. 11 wild provenances collected.*

Response: What we want to convey here is that the 11 varieties originated from four provinces in eastern China. To facilitate understanding, we have also revised the expressions in the method section：

From September to October 2022, wild P. cyrtonema rhizomes were collected from 11 provenances across four provinces (Anhui, Zhejiang, Fujian, and Jiangxi) in China (Fig 1). Ninety individual plants were sampled from each provenance, resulting in a total of 990 rhizome samples. Geographical coordinates and altitude for each collection site were recorded in detail (Table 2). All plant materials were authenticated as *P. cyrtonema* by Professor Shi-Ming Cheng of the Zhejiang Academy of Forestry. Each collected rhizome, containing two nodes and one bud (equivalent to a two-year-old growth unit), served as a planting unit. These units were temporarily heeled-in in a greenhouse nursery at the Zhejiang Academy of Forestry prior to the formal experiment. **It has been added in new line 111-120.**

Comments: *Without much clarity on the samples collected from how many provenances, it is very unclear to understand. If authors collected 300 rhizomes per provenances, then it 3,300 rhizomes. Do the authors planted all 3300 rhizomes? If so, why authors only chose 30 for further analysis? The whole sample collection and planting details and sample selection strategies for further analysis is completely confusing.*
 Response: We revised the relevant descriptions in the method. In fact, for each provenance, 90 samples were collected for the experiment. There were 3 experimental plots in each location, and 10 samples in each plot:

From September to October 2022, wild *P. cyrtonema* rhizomes were collected from 11 provenances across four provinces (Anhui, Zhejiang, Fujian, and Jiangxi) in China (Fig 1)……. In November 2023, rhizomes of all provenances were excavated from the three trial sites and transported to the laboratory for processing. The rhizomes were excised, thoroughly cleaned, and then dried in an oven at 70°C until a constant weight was achieved. The dry weight was measured, and the samples were subsequently used for medicinal component analysis. The polysaccharides, saponins, total phenols and flavonoids, in *P. cyrtonema* rhizome were determined using kits (Suzhou Comin Biotechnology Co., Ltd., Suzhou, China) in accordance with the kits’ instructions [27]. **It has been revised in new lines 111-145.**

Comments: *Authors talk about “Polysaccharides, saponins, total phenolics, and flavonoids were quantified using commercial assay kits” which is completely unacceptable at this current format. Authors provide elaborate details of how this was carried out.*

Response: The polysaccharides, saponins, total phenols and flavonoids, in P. cyrtonema rhizome were determined using kits (Suzhou Comin Biotechnology Co., Ltd., Suzhou, China) in accordance with the kits’ instructions. **It has been added in new lines 138-162.**

The total polysaccharide content was determined using the phenol-sulfuric acid method: approximately 0.05 g of dried sample was weighed, extracted with hot water, and subjected to alcohol precipitation. The supernatant was reacted with phenol-concentrated sulfuric acid reagent at 90°C for 20 minutes, and the absorbance was measured at 490 nm. Glucose was used as the standard for quantification. The total phenol content was determined by the Folin-Ciocalteu method: approximately 0.02 g of dried sample was extracted with 60% ethanol and then reacted with tungstic acid reagent at 25°C for 10 minutes. The absorbance was measured at 760 nm, and gallic acid was used as the standard for calculation. The flavonoid content was measured using the aluminum nitrate colorimetric method: the sample extract was reacted with alkaline aluminum nitrate reagent at 25°C for 15 minutes, and the absorbance was measured at 510 nm. Rutin was used as the standard for calculation. The total saponin content was determined by the vanillin-perchloric acid method: after ultrasonic extraction and evaporation to dryness, the sample was reacted with the coloring reagent at 55°C for 20 minutes, and the absorbance was measured at 589 nm. Oleanolic acid was used as the standard for calculation. All contents were expressed per gram of dry weight (mg·g^-1^).

Comments: *Whether whole plant is used to quantify the chemical compounds or only leaf and so on details are missing.*

Response: In the original manuscript, we already mentioned that this research utilized plant rhizomes for the analysis experiments: In November 2023, rhizomes of all provenances were excavated from the three trial sites and transported to the laboratory for processing. The rhizomes were excised, thoroughly cleaned, and then dried in an oven at 70°C until a constant weight was achieved. The dry weight was measured, and the samples were subsequently used for medicinal component analysis. The polysaccharides, saponins, total phenols and flavonoids, in *P. cyrtonema* rhizome were determined using kits (Suzhou Comin Biotechnology Co., Ltd., Suzhou, China) in accordance with the kits’ instructions [27]. **As shown in new lines 138-145.**

Comments: *Authors indicating alphabets in table 3 and 4 is very confusing and unable to read the table. I have no idea why authors wanted alphabets to represent values.*

Response: We have re-performed the data analysis and re-arranged the data. Please refer to Table 4 (the new version).

Comments: *Line 181-188: Very confusing statement. I suggest authors break the long sentences for better understanding.*

Response: We sincerely apologize that the previous statement may have caused confusion to you. We have conducted a re-analysis of the data, and the details can be found in lines 205 to 238.

Comments: *If authors have photographs of the* *rhizomes, plants were grown in experimental set up and other relevant photographs of plants can be provided in the article*

Response: We added photos of the rhizomes and the experimental field in Figure 1.

Please review our manuscript again. If you have any further queries, please do not hesitate to contact us.

Sincerely yours

Xiaodeng Shi

Nov. 18, 2024

*Zhejiang Academy of Forestry, Hangzhou, China.*
