# Supplementary material for: Variation analysis of growth traits and medicinal components in different provenances of Polygonatum cyrtonema based on heterogeneous garden experiment
Source: PLoS One. 2026 Apr 10;21(4):e0346920. doi: 10.1371/journal.pone.0346920 (PMC13068254; doi:10.1371/journal.pone.0346920)
Supplement: Attachment — Submitted filename: Response to reviewers.docx [file pone.0346920.s004.docx]

Dear editors and reviewers.

Thank you for your kind letter about my manuscript “Variation analysis of growth traits and medicinal components in different provenances of *Polygonatum cyrtonema* based on heterogeneous garden experiment” (PONE-D-25-60346) on Jan. 19. 2026. We would like to express our great appreciation to you and the reviewers for patient work on our paper. We have finished revising the manuscript and would like to resubmit it again.

We revised the manuscript in accordance with the reviewers’ comments, and carefully proof-read the manuscript. Here below is our description on revision according to the reviewers’ comments.

**Journal Requirements:**

1. Comments: *Please ensure that your manuscript meets PLOS ONE's style requirements, including those for file naming.*

Response: We adjusted the overall format of the manuscript (including the title page) to ensure that the manuscript format complies with the style requirements of PLOS ONE.

2. Comments: *In your Methods section, please provide additional information regarding the permits you obtained for the work. Please ensure you have included the full name of the authority that approved the field site access and, if no permits were required, a brief statement explaining why.*

Response: The three experimental garden sites (Longyou, Jinyun, and Lin‘an) are long-term, dedicated research bases established and managed by our host institution, the Zhejiang Academy of Forestry. These sites are used exclusively for scientific trials by our research team. Therefore, no specific permits from external authorities were required for accessing and conducting research at these institutional facilities.

We have updated the “Materials and Methods” section (Section 2.1) accordingly to include this statement. “The field studies did not require specific permits for the experimental sites, as all three locations (ZJLY, ZJJY, and ZJLA) are long-term research bases owned and managed by the Zhejiang Academy of Forestry for scientific purposes.” in the revised line 134-137.

3. Comments: *Please state what role the funders took in the study.*

Response: This study was supported by the [Pioneer and Leading Goose +X” R&D Program of Zhejiang(2024C04007)]. Prof. Shiming Cheng, as the recipient of this funding project and the first author of this manuscript, was responsible for conceptualization, methodology, supervision, project administration.

4. Comments: *Please remove any funding-related text from the manuscript and let us know how you would like to update your Funding Statement.*

Response: We removed the Funding Statement from the manuscript.

5 Comments: *In the online submission form, you indicated that [The data underlying the results presented in the study are available from corresponding authors].*

*All PLOS journals now require all data underlying the findings described in their manuscript to be freely available to other researchers, either 1. In a public repository, 2. Within the manuscript itself, or 3. Uploaded as supplementary information.*

*This policy applies to all data except where public deposition would breach compliance with the protocol approved by your research ethics board. If your data cannot be made publicly available for ethical or legal reasons (e.g., public availability would compromise patient privacy), please explain your reasons on resubmission and your exemption request will be escalated for approval.*

Response: In accordance with PLOS's requirements, we confirm that all data underlying the findings reported in this manuscript are fully available within the manuscript itself.

Specifically, the key quantitative data are presented in the following tables and figures:

The core phenotypic and medicinal component data for all 11 provenances across three sites are provided in Table 4 and Table 5.

The results of the variance and correlation analyses are detailed in Figure 2.

The principal component analysis scores and the clustering results that support the main conclusions are shown in Table 6, Table 7 and Figure 3.

6. Comments: *PLOS requires an ORCID iD for the corresponding author in Editorial Manager on papers submitted after December 6th, 2016. Please ensure that you have an ORCID iD and that it is validated in Editorial Manager. To do this, go to ‘Update my Information’ (in the upper left-hand corner of the main menu), and click on the Fetch/Validate link next to the ORCID field. This will take you to the ORCID site and allow you to create a new iD or authenticate a pre-existing iD in Editorial Manager.*

Response: We have updated the ORCID information of the corresponding author in Editorial Manager.

7. Comments: *We note that Figure 1 in your submission contains map images which may be copyrighted.*

Response: We would like to clarify that the map in Figure 1 is an original schematic map created by us solely for the purpose of illustrating the geographical locations of the *P. cyrtonema* provenances and experimental sites described in the study.

The map was generated using publicly available geographic coordinate data and was produced with professional drawing software (ArcGIS). It does not contain or derive from any copyrighted map material, such as Google Maps, Google Earth, or other proprietary sources. Therefore, we confirm that this figure is free of third‑party copyright restrictions and is fully compatible with the CC BY 4.0 license.

To avoid any ambiguity, we are happy to update the figure caption as follows:

“Figure 1. Schematic map showing the locations of P. cyrtonema provenances and experimental sites. The map was created by ArcGIS using geographic coordinates and is intended for illustrative purposes only.” It has been added in the revised line 145-146.

**Reviewer #1**

Comments: *Resource depletion was cited in the introduction, but it does not quantify the declines of wild P. cyrtonema populations (e.g. IUCN status, harvest volumes); please provide evidence as to why it is urgent for provenance trials to be conducted in the first place, as compared to other conservation initiatives.*

Response: We have revised the introduction to provide a clearer and evidence-based rationale. The urgency for conducting this provenance trial is twofold:

Quantified Conservation Status: As correctly noted, *P. cyrtonema* is classified as Near Threatened (NT) on the IUCN Red List. This formal, global assessment provides concrete evidence of population declines and the risk of over-exploitation, directly justifying research aimed at reducing wild harvest pressure.

Addressing the Cultivation Bottleneck: Provenance trials are a critical and specific step within conservation strategies. While habitat protection (in-situ) and seed banking are vital, they do not solve the immediate problem of cultivation failure due to poor-performing, unselected germplasm. Our trial identifies high-yield, high-quality provenances (e.g., Songyang, Yunhe). This enables the establishment of productive and economically viable cultivation, which is the most direct way to provide a sustainable alternative to wild collection and alleviate pressure on NT wild populations.

In the revised manuscript, we cite the IUCN status and reframe our introduction to emphasize that provenance selection is the essential first link in the chain toward effective conservation-through-cultivation.

“According to the IUCN Red List of Threatened Species, P. cyrtonema is classified as Near Threatened (NT) [15], which confirms the ongoing decline of its wild populations and the risk of over-exploitation. Thus, it is in a critically endangered state and is in urgent need of protection. Artificial introduction and cultivation are effective ways to protect and preserve germplasm resources of endangered plants [16]. Introduction and cultivation require an understanding of the environmental conditions that affect its growth and the selection of suitable habitat areas to be successful [17]. For medicinal plants, the initial and fundamental step is to screen provenances with high contents of bioactive compounds for domestication and cultivation through provenance trials. This approach is essential to establish a productive and economically viable cultivation model, thereby facilitating scaled-up production.” It has been added in the revised line 78-88.

Comments: *Considering the earlier single-site investigations conducted on floral phenotypes/minerals, how exactly does the current multi-environment design fill the already identified research gaps in GxE interactions for the coordinated analysis of growth-medicinal traits?*

Response: Our multi-environment design fundamentally advances the understanding of G×E interactions for coordinated trait analysis in three key ways that a single-site study cannot:

Quantifies G×E Interaction: In the Table 3, we provide the first formal statistical evidence (significant Provenance × Site interaction, P < 0.01) that the performance of provenances in both growth and medicinal traits is environment-dependent. A single-site study cannot detect this crucial interaction.

Identifies Stable Superior Germplasm: It distinguishes locally adapted provenances from those that are consistently superior across diverse environments. Identifying Songyang and Yunhe as top performers in all three sites is a key, reliable outcome only possible through a multi-site design.

Reveals Environmental Robustness of Trait Relationships: Most importantly, it tests whether the coordination between growth and medicinal traits is itself stable across environments. We found that the strong positive correlations between key growth traits and major medicinal components (e.g., polysaccharides, flavonoids) were consistently maintained across all trial sites. This indicates that the genetic or physiological linkage driving this synergy is robust to the environmental variation tested, a finding critical for breeding reliable cultivars.

In summary, our design moves from describing static trait associations to analyzing the environmental stability of these associations, directly filling the identified gap for coordinated growth-medicinal trait breeding.

Comments: *While the discussion recommends the SY/YH provenances for breeding, the very high GxE interactions (marked differences in P×S effects across all traits) would suggest that these are adapted to specific sites; how should breeders make their recommendations with regard to stability versus mean performance?*

Response: We agree that significant G×E interaction necessitates careful breeding recommendations. Our endorsement of the SY and YH provenances is based not only on their superior mean performance but crucially on their consistent top-rank stability across all three test sites. This indicates a stable, high-performing genetic potential across diverse conditions rather than narrow adaptation to a single site.

For breeders, we provided practical recommendations in the discussion. Specifically, lines 459-475 suggest that Songyang and Yunhe provenances, demonstrating significant adaptive advantages across all three trial sites, can be cultivated as superior germplasm, while TG and JJ provenances can be developed as specialized lines for high saponin content.

Comments: *Saponin accumulation is altitude/AMT correlated but growth uncorrelated; offers on possible biosynthetic route or stress reactions explaining this plus if any conflicting viewpoints fasten or limit "ideal" provenance selection for the understory economy.*

Response: In this study, we did not find any viewpoints that contradicted those of previous research. In the discussion (Line 412-422), we explained the reasons why the accumulation of saponins is related to altitude and the annual average temperature:“ In comparison, saponin and flavonoid accumulation in *P. cyrtonema* demonstrated clear environmental drivers. Higher altitude, warmer climate, and lower latitude and longitude were found to favor saponin accumulation, consistent with studies on *Paris polyphylla* [48] and *Bupleurum chinense* [49]. The Jinyun experimental site in this study showed significantly higher saponin content than other sites. Located in the western mountainous area of Zhejiang Province with complex terrain, significant temperature variations, and uneven seasonal precipitation distribution, Jinyun's environmental conditions support the hypothesis that such stress factors stimulate the synthesis of defensive secondary metabolites like saponins [50]. Our results strongly support this theory, indicating that saponins may serve as key chemical defense compounds in *P. cyrtonema*'s adaptation to specific alpine environmental stresses.”

At the same time, we also pointed out that both plant height and saponin content showed positive responses to annual temperature, suggesting the potential for synergistic enhancement of growth and saponin accumulation in warm regions, providing valuable screening clues for developing high-yield, high-quality varieties (Line 435-440).

Comments: *High heritability estimates (h²=0.63-0.99) are given indicating good genetic control, yet due to low degree of replication (30/site/provenance), an overestimation might take place; how does this compare to similar medicinal plant trials (Dendrobium, Astragalus)?*

Response: We agree that replication can influence heritability (h²) estimates. We believe our multi-site design addresses this and our estimates are reliable:

While within-site replication is 30, our three distinct environments provide crucial "environmental replication." The joint analysis across sites allows for more robust variance partitioning than a single site with higher replication, a standard method in perennial plant breeding.

Our heritability range (0.63–0.99) and scale (11 provenances) are consistent with foundational trials in comparable medicinal plants (e.g., Dendrobium, Astragalus), where similar designs are used to estimate genetic parameters and guide selection.

Biological Validity: The traits with the highest h² (e.g., height) showed the most stable rankings across all three sites, confirming their strong genetic control. The provenances we recommend (e.g., Songyang) were consistently superior across environments, not due to chance.

We will add a brief statement in the Discussion：Although environmental effects significantly affected trait expression across different sites in our study, the provenance repeatability remained notably high (0.63-0.99). This pattern shows similarities to observations in other medicinal plants, such as Dendrobium officinale [42]and Astragalus mongolicus[43], indicating substantial heritability for these traits and suggesting significant potential for genetic gain through selection [44]. It has been added in the revised line 360-361.

Comments: *Were required field permits secured to collect wild rhizomes from the eleven provenances in four provinces?*

Response: We confirm that all rhizome samples were obtained in full compliance with research norms and through legitimate channels.

Specifically, the collections within Zhejiang Province were conducted from our institution’s (Zhejiang Academy of Forestry) own experimental fields or long-term observation plots, which are managed by our research team for scientific purposes. For collections from the other three provinces (Anhui, Fujian, and Jiangxi), the materials were acquired through formal germplasm exchange agreements with local partners in those regions. These exchanges were carried out for mutual non-commercial, scientific research purposes.

Comments: *Soil physicochemical properties (pH, NPK, organic matter) and microbiome profiles were not measured at the three sites; how might unaccounted edaphic/microbial factors confound the reported GxE interactions?*

Response: We fully agree with the reviewer that understanding the specific role of soil and microbiome is a fascinating and important next step. In our Discussion (in the line 482-487), we have acknowledged this limitation: “(2) Complex field factors (soil types, microbiome communities) were not systematically quantified [35], possibly constraining the generalizability of conclusions. Future work should expand to representative ecoregions (e.g., southwestern mountains, North China Plain), employing integrated transcriptomic-metabolomic analyses to decipher "gene-environment-metabolite" regulatory networks.”

However, we would like to clarify why we believe these unmeasured factors do not confound the core conclusions of our study regarding provenance performance and adaptation patterns:

(1) The primary goal of our multi-site trial was not to attribute variation to individual soil parameters, but to test provenance performance under real-world, integrated environmental complexes (climate + soil + microbiome). The significant “Site” effect confirms that the three locations provided meaningfully different growing conditions, which include their inherent but uncharacterized edaphic differences. Therefore, the G×E interactions we report are a robust measure of how provenances respond to these composite site-specific conditions, which is precisely what matters for practical breeding and deployment recommendations.

(2) The most pronounced geographic correlations we found (e.g., saponins with altitude/temperature, flavonoids with rainfall) align well with known macro-climatic stressors (e.g., annual average temperature, annual rainfall) that drive secondary metabolism. While soil can modulate these responses, it is less likely to be the primary driver of such clear, large-scale clinal patterns. This consistency supports the interpretation that the observed G×E patterns have a strong climatic basis.

(3) Crucially, our main recommendation of provenances like Songyang and Yunhe is based on their consistent superiority across all three sites. If their performance were heavily confounded by unmeasured, site-specific soil factors, we would expect them to excel in only one location, not all. Their stability across environments with likely varying soils strengthens the case for their genetic superiority and broad adaptability.

Comments: *High values of PCV/GCV (>40% for some traits like polysaccharides) and almost perfect repeatability (h²=0.99) are reported; please discuss potential overestimation due to low replication (n=10 per provenance-block).*

Response: We acknowledge the concern that limited within-block replication (n=10) could, in theory, lead to an overestimation of variance components. However, we contend that our multi-site common garden design provides a robust framework to mitigate this issue and that the high estimates, particularly for key traits, reflect genuine biological significance. Specifically, our three environmentally distinct trial sites provided essential higher-order replication; the joint analysis across these locations allows for a more reliable partitioning of genetic and environmental variance than could be achieved within a single site. The high phenotypic and genotypic coefficients of variation (PCV & GCV >40%) were most pronounced for polysaccharides. This is of particular biological and pharmacological relevance, as polysaccharides are not only the primary bioactive constituents of *P. cyrtonem*a but also the sole component specified for content limitation in its monograph in the Chinese Pharmacopoeia. The extreme genetic variability we observed for this definitive quality trait underscores its strong potential for selective breeding. Furthermore, the near-perfect repeatability estimates are corroborated by the consistent superior performance of provenances like Songyang and Yunhe across all three disparate environments—a pattern unlikely if the high heritability were merely a statistical artifact of low replication.

Comments: *Provenances SY and YH ranked highest by means of PCA; however, saponin content (key bioactive) was lower in these; does this imply trade-offs in breeding for "high-quality" versus specific compounds, and how were the scoring parameters weighted?*

Response: You are correct that the SY and YH provenances, while ranked highest in our PCA-based, did not exhibit the absolute highest saponin content. This does indeed reflect a central consideration in breeding. Our PCA model assigned weights based on the contribution of each trait to overall variance, aiming to identify provenances with superior comprehensive performance across the interconnected spectrum of growth vigor (e.g., plant height, stem diameter), yield-related traits (e.g., leaf area), and multiple key medicinal components (polysaccharides, flavonoids, total phenolics). Consequently, SY and YH were selected for their outstanding and balanced performance across this suite of economically important traits, which is crucial for developing a commercially viable cultivar where overall biomass and a broad profile of bioactive compounds determine economic value. Importantly, their saponin content was not low but intermediate (ranking mid-tier among the 11 provenances) and, based on literature values for *P. cyrtonem*a saponins which typically range from approximately 8.57 to 50.1 mg/g (Li et al., 2023; Wang et al., 2020), falls well within the recognized bioactive range. This suggests they possess perfectly adequate saponin levels for medicinal use. Their selection implies a pragmatic breeding strategy that prioritizes overall agronomic and medicinal quality while maintaining satisfactory levels of specific compounds, rather than maximizing a single component at the potential expense of others like yield. For a breeding program specifically targeting ultra-high saponin content, one would adjust the selection indices to heavily weight that single trait, potentially selecting a different provenance like TG. Thus, our results delineate a clear strategy: SY and YH serve as elite, well-rounded founders for general cultivar development, while other provenances serve as specialized donors for trait-specific breeding. (Line 459-475)

Li, L.H., Deng, Y.Q., Tian, Y.Q., et al. Comparative of main components of *Polygonatum* from different habitats based on clustering and principal component analysis. MMJC. 2023, 25(9):10-18.

Wang, D., Zhang, H., Liu, J.L., Man, Q., Dong, X.R., et al. Comparative Analysis of Bioactive Components of *Polygonatum cyrtonema* Hua from Different Areas. Agricultural Science ＆ Technology, 2020, 21(4): 50-57.

Comments: *The correlation analysis shows saponins positively correlated to altitude/AMT and uncorrelated to growth traits; suggest biological considerations (e.g., stress-induced saponogenesis) that can complement literature on these geo-climatic variables.*

Response: We agree that the observed pattern—saponins being positively correlated with altitude/mean annual temperature but uncorrelated with growth traits—strongly points towards an underlying biological mechanism rather than a simple co-variation. We appreciate the suggestion to discuss stress-induced saponogenesis (the biosynthesis of saponins under stress).

During our discussion, we also revealed the essence of this phenomenon, as shown in lines 413 to 420：“Higher altitude, warmer climate, and lower latitude and longitude were found to favor saponin accumulation, consistent with studies on *Paris polyphylla* [53] and *Bupleurum chinense* [54]. The Jinyun experimental site in this study showed significantly higher saponin content than other sites. Located in the western mountainous area of Zhejiang Province with complex terrain, significant temperature variations, and uneven seasonal precipitation distribution, Jinyun's environmental conditions support the hypothesis that such stress factors stimulate the synthesis of defensive secondary metabolites like saponins [55].”

**Comments:** *Prior studies (e.g., Jiang et al. 2022 on mineral elements) exist on P. cyrtonema provenances; what unique advances does this multi-site trial offer beyond single-environment work, especially for understory economy applications?*

Response: Thank you for this excellent question, which allows us to clarify the pivotal conceptual and practical advancements of our multi-site common garden trial beyond valuable prior single-environment studies, such as the work on mineral elements by Jiang et al. (2022). While single-site provenance evaluations are fundamental for documenting the existence of phenotypic and compositional variation, our design uniquely addresses the critical question of stability and adaptability across heterogeneous environments, which is the core of effective application in the understory economy. The primary unique advance is the direct quantification of Genotype-by-Environment (G×E) interaction, evidenced by the highly significant Provenance × Site effects we report. This statistically proves that the performance ranking of provenances is not fixed but changes with location. Consequently, our study moves beyond providing a static "performance ranking" valid only for one site to delivering a dynamic "adaptability portfolio." We can now distinguish between broadly adapted, stable superior provenances (e.g., Songyang and Yunhe, which excelled across all three sites) and specifically adapted ones. This fundamentally de-risks the recommendation process for forest farmers: instead of receiving a single list that may fail in their specific local context, they gain access to differentiated recommendations—stable all-rounders for reliable yield or specialized provenances for targeting specific bioactive compounds in their particular geo-climatic setting.

For the understory economy, these advances translate into direct, actionable value. First, identifying stable provenances maximizes planting success and economic resilience for growers across diverse locations, providing a safer foundation for scaling up cultivation. Second, by mapping key medicinal components (like saponins and flavonoids) to specific environmental drivers (altitude, temperature, rainfall), our study enables precision sourcing. Communities can leverage their specific local conditions (e.g., higher altitude) to cultivate provenances predisposed for higher concentrations of targeted compounds, fostering development of niche, value-added products and avoiding homogeneous competition. Therefore, the unique contribution of this multi-site trial lies in shifting the paradigm from merely identifying potential in one place to predicting performance across many, thereby providing the essential ecological and genetic toolkit needed for the spatially optimized and sustainable development of P. cyrtonema cultivation in diverse forest landscapes.

We added the discussion in the line 479-486: “This multi-site trial advances prior single-environment work by directly quantifying Genotype-by-Environment (G×E) interactions. This enables a critical distinction between broadly adapted provenances, which ensure reliable yield across diverse sites, and specifically adapted ones optimized for particular conditions or target compounds. Consequently, it provides an ecological-genetic basis for spatially precise cultivation strategies within the heterogeneous understory economy, moving beyond mere cataloging of variation toward predictive and de-risked application.”

**Reviewer #2:**

**Comments: (Line 23–24):** *The authors have mentioned high phenotypic and genotypic variance for the studied traits (in abstract); however, Table 4 presents a range of low, moderate, and high GCV and PCV values across different traits. This discrepancy should be clarified to ensure consistency between the text and the presented data.*

Response: In the abstract, we used the term “high” in a relative sense, based on common benchmarks in provenance trials and breeding studies for medicinal plants, where phenotypic and genotypic coefficients of variation (PCV and GCV) exceeding 10–15% are generally considered to indicate moderate to substantial genetic variability and potential for selection. As shown in Table 4, while certain traits such as saponins consistently exhibited lower PCV values (ranging from 7.41% to 10.12%), the majority of traits—particularly polysaccharides (PCV up to 46.89%), plant height, leaf area, and flavonoids—displayed notably higher variability across the three trial sites. For instance, polysaccharide content showed PCV values of 46.77%, 45.29%, and 41.24% at the ZJLY, ZJJY, and ZJLA sites, respectively, which indeed represent high levels of variation in the context of secondary metabolite accumulation in perennial herbs.

To ensure consistency and avoid any potential misunderstanding, we will revise the relevant phrasing in the abstract and the results section to more precisely reflect the spectrum of variability observed. Specifically, in the abstract, we will modify the sentence to read: “Substantial phenotypic (PCV: 7.41%–46.89%) and genotypic (GCV: 5.99%–44.92%) coefficients of variation were observed, with particularly high variation in polysaccharides and key growth traits.” It has been revised in the line 23-24.

**Comments: (Line 45–47):** *The meaning of the sentence “Its dried rhizome, designated as ‘Polygonati Rhizoma’ in the Chinese Pharmacopoeia (ChP), is one of the three primary botanical sources for this official medicinal material” is unclear. The authors should explicitly specify which official medicinal material is being referred and which three primary botanical sources.*

Response: In the cited sentence, the "official medicinal material" refers specifically to the crude drug known as "Huangjing" or "*Polygonati Rhizoma*" as standardized in the Chinese Pharmacopoeia (ChP). According to the current edition of the ChP, the official monograph "*Polygonati Rhizoma*" lists three primary botanical sources: the rhizomes of *Polygonatum cyrtonema* Hua (the species studied in this manuscript), *Polygonatum sibiricum* Red., and *Polygonatum kingianum* Coll. et Hemsl. Therefore, the sentence intends to convey that the dried rhizome of *P. cyrtonema* is one of the three officially recognized species used to produce the medicinal material "*Polygonati Rhizoma*." To eliminate any ambiguity in the revised manuscript, we will rephrase the relevant sentence to explicitly state: "Its dried rhizome, designated as 'Polygonati Rhizoma' in the Chinese Pharmacopoeia (ChP), is one of the three primary botanical sources (together with *Polygonatum sibiricum* and *Polygonatum kingianum*) for this official medicinal material." It has been added in the revised line 48-50.

**Comments: (Line 52–57):** *needs to simplify the complex sentences like “As one of the most promising medicinal and edible plants, P. cyrtonema holds a strategic position in the traditional Chinese medicine industry and the forest wellness economy, underscoring its significant economic and industrial value as a key understory economic crop in southern China”*

Response: In the revised manuscript, we will simplify and restructure the sentence for greater clarity. A proposed revision is: "As a prominent medicinal and edible plant, *P. cyrtonema* holds strategic value in both the traditional Chinese medicine industry and the forest wellness economy. It is therefore considered a key understory economic crop in southern China, with significant industrial and economic potential." **It has been added in the revised line 57-60.**

**Comments:** *The authors have not addressed the appropriate harvesting period after planting. As many Polygonatum species require 2–3 years to reach optimal harvest maturity, this information is crucial, as the harvesting stage can substantially influence both biomass accumulation and the yield of active medicinal constituents. Therefore, the authors should provide a clear and appropriate justification for evaluating medicinal components after only one year of growth.*

Response: We fully acknowledge that for many *Polygonatum* species, including *P. cyrtonema*, the accumulation of rhizome biomass and secondary metabolites is a multi-year process, and the optimal harvest period for commercial production is indeed typically after 2–3 or more years of growth.

However, in this study, our primary objective was not to determine the optimal agronomic harvest time for maximum yield, but rather to conduct a provenance trial aimed at evaluating and comparing the relative performance of different genetic sources (provenances) under standardized, multi-environment conditions. Such trials are fundamentally designed for early-stage genetic selection and often assess traits at a defined, comparable stage—even if it is not the final commercial harvest point. The one-year growth period was selected as a standardized, practical timeframe for the initial screening of a large number of provenances (11 sources across 3 sites) to identify promising genetic material with superior potential. This approach is common in forest tree and perennial plant breeding programs, where early growth traits and juvenile-mature correlations are used for preliminary selection to improve efficiency and reduce lengthy field trial durations.

The core premise of our approach is that significant genetic variation and consistent performance rankings among provenances for key traits can be detectable even at an early developmental stage. Our data strongly support this: we observed highly significant (P < 0.01) differences among provenances for all growth and medicinal traits after just one year (Table 3), with high repeatability (Table 4), indicating clear genetic differentiation. This provides a robust basis for preliminary selection of promising genetic material for further breeding and longer-term evaluation. Nonetheless, we explicitly acknowledge in the manuscript (as noted in the Study Limitations section) that our findings are based on a single harvest point and that longer-term trials are needed to confirm the stability and maturation of these traits.“To assess their adaptive evolution and establish a sustainable utilization framework, future work should conduct long-term monitoring of different *P. cyrtonema* provenances under climate change scenarios, which must include systematic yield estimation.”In the line 499-502.

**Comments:** *The authors have included growth traits and leaf dimensions together with PCV, GCV, and P²h values in Table 4. However, variability parameters such as PCV, GCV, and P²h are generally expressed in percentages or as unitless values, rather than in physical units (e.g., cm or mg). Therefore, it would be more appropriate to present these variability parameters in a separate table to improve clarity and avoid confusion.*

Response: We agree that presenting measured trait values (with units such as cm or mg·g⁻¹) together with unitless variability parameters (PCV, GCV, and *h*²P as percentages or proportions) in the same table may reduce immediate clarity for readers, as the two types of data serve different interpretive purposes—the former indicating trait magnitude, and the latter reflecting the extent and heritability of variation.

In response to your suggestion, we will restructure the presentation of these results in the revised manuscript. Specifically, we propose to split the current Table 4 into two separate tables.

Table 4. Analysis of growth traits among provenances of *P. cyrtonema* across experimental sites

Table 5. The PCV, GCV and h2P values of various traits in different experimental sites

**Comments:** *Rhizome biomass yield is a critical trait for Polygonatum species, both in terms of growth performance and economic value. Although the authors mention the estimation of dry biomass in the methodology, this parameter has not been included in the analysis or results.*

Thank you for raising the important point about rhizome biomass yield. In this provenance trial, our primary focus was the comparative screening of genetic material for key quality traits (medicinal compound concentration) and early growth form. This is an established approach for initial germplasm selection, distinct from later-stage agronomic yield trials.

We have revised the manuscript to clearly acknowledge this scope. In the Discussion, we now state: “To assess their adaptive evolution and establish a sustainable utilization framework, future work should conduct long-term monitoring of different *P. cyrtonema* provenances under climate change scenarios, which must include systematic yield estimation.” **It has been added in line 499-502.**

**Comments:** *please check font size and type (line 303) it should be uniform throughout the manuscript.*

Response: Thank you for your attentive review and for pointing out the inconsistency in font size and type at line 303. We sincerely apologize for this oversight in formatting. In our revised manuscript, we will conduct a thorough check of the entire document to ensure complete consistency in font type, size, and style for all text elements

**Comments:** *I think there is scope to analyze stability in yield of rhizome biomass and medicinal comports across different trial sites. Eberhart & Russell Model; AMMI (Additive Main Effects and Multiplicative Interaction) or GGE Biplot analysis can be used.*

Response: We agree that these are powerful and established methods (Eberhart & Russell, AMMI, or GGE biplot) for visualizing genotype-by-environment interaction (G×E) patterns and for identifying genotypes with broad or specific adaptability in multi-environment trials.

In the present study, our primary objective was to conduct an initial, comparative screening of multiple *P. cyrtonema* provenances across heterogeneous environments to identify promising genetic material for breeding. To achieve this, we focused on quantifying the extent of variation and the major patterns of differentiation using a combination of analytical approaches. The joint analysis of variance (ANOVA) explicitly tested and confirmed the highly significant effects of provenance, site, and their interaction (G×E) for all traits (Table 3), which establishes the fundamental premise for stability analysis. Subsequently, principal component analysis (PCA) was employed to reduce the dimensionality of the multi-trait dataset and to integrate the key growth and medicinal components into a few composite indicators. This allowed us to objectively rank the provenances based on their overall performance. Hierarchical cluster analysis further grouped the provenances into distinct performance categories (Fig. 3). Together, these methods effectively addressed our core research aims: to quantify variation, identify key environmental drivers, and select superior provenances for Eastern China.

We believe that the current analytical framework is sufficient and appropriate for the stated goals of this first multi-site provenance evaluation. The significant G×E interaction revealed by ANOVA, combined with the clear provenance rankings and classifications from PCA and cluster analysis, provides a robust and interpretable basis for our primary conclusions and breeding recommendations.

However, we fully acknowledge the reviewer's insightful point that dedicated stability analysis could offer an additional layer of interpretation, particularly for visualizing the specific nature of interactions and for defining ideal test environments. In response, we are happy to enhance the discussion by incorporating a statement that recognizes the potential of such methods for future, more detailed studies. Specifically, we will add a sentence in the Discussion (likely in section 4.4 "Study Limitations") along these lines (493-496):
"Although the current study employed combined ANOVA and PCA to identify superior provenances, future multi-environment trials could adopt stability analysis approaches such as AMMI or GGE biplot to select provenances with broad or specific adaptability. "

Please review our manuscript again. If you have any further queries, please do not hesitate to contact us.

Sincerely yours

Xiaodeng Shi

Jan. 30, 2026

*Zhejiang Academy of Forestry, Hangzhou, China.*
