# Supplementary material for: Variation analysis of growth traits and medicinal components in different provenances of Polygonatum cyrtonema based on heterogeneous garden experiment
Source: PLoS One. 2026 Apr 10;21(4):e0346920. doi: 10.1371/journal.pone.0346920 (PMC13068254; doi:10.1371/journal.pone.0346920)
Supplement: Attachment — Submitted filename: Response_to_reviewers_auresp_2.docx [file pone.0346920.s005.docx]

Dear editors and reviewers.

Thank you for your kind letter about my manuscript “Variation analysis of growth traits and medicinal components in different provenances of *Polygonatum cyrtonema* based on heterogeneous garden experiment” (PONE-D-25-60346) on Feb. 23. 2026. We have carefully addressed all comments and suggestions in this minor revision. The modifications include:

1. Comments: *Heritability estimates are very high (up to 0.99). While the authors defend this via multi-site replication, a brief note on potential limitations or comparisons to other perennial medicinal plants in the results/discussion would strengthen the interpretation.*

Response: Thank you for this thoughtful observation regarding the high heritability estimates reported in our study (up to 0.99). In the revised manuscript, we have included comparisons with **heritability data** estimates from other understory medicinal plants (***Panax ginseng* (0.63–0.96)*, Picrorhiza kurrooa*(0.65-0.86)**) to further corroborate the robustness of our findings, while also acknowledging the limitations of the current study:

“Although environmental effects significantly affected trait expression across different sites in our study, the provenance repeatability remained notably high (0.63-0.99). This pattern shows similarities to observations in other medicinal plants, such as *Panax ginseng* (0.63–0.96) [42] and *Picrorhiza kurrooa* (0.65-0.86) [43], indicating substantial heritability for these traits and suggesting significant potential for genetic gain through selection [44].” **It has been revised in the line 359-361.**

“Nevertheless, we acknowledge that these estimates are derived from a single-year assessment. Multi-year longitudinal studies would be valuable to confirm the stability of genetic control across different developmental stages and to account for potential temporal variation in trait expression.” **It has been added in the line 366-369.**

We have changed the reference.

[42] Zhao, S.J., Li, F.Y., Zhao, Y.H., et al. Studies on Selection Theory for High-yield Variety of Panax ginseng and Breeding of Jishen 1. *Sci. Agr. Sin.* 1998, 31(5).

[43] Hemdan, A., Chauhan, R.S., Pant, S.C. et al. Variability and heritability for yield and yield attributing characters in an endangered medicinal herb Picrorhiza kurrooa Royle ex Benth under cultivation in Bharsar, Uttarakhand, Western Himalaya, India. *Genet. Resour. Crop. Evol.*2025*,* 72, 8079–8099.

2. Comments: *The one-year harvest is justified for early screening, but the economic implications of longer-term yield (biomass) should be more explicitly discussed in the context of understory farming.*

Response: In response to your suggestion, we have expanded the Discussion section 4.4 to more explicitly address the economic implications of longer-term yield in the context of understory farming systems.

“While the one-year harvest in this study serves as an effective early screening strategy for identifying genetically superior provenances, it is important to note that the full economic potential of these selections in understory farming systems can only be realized through multi-year cultivation. In practice, *P. cyrtonema* is typically harvested after 2–3 years to maximize rhizome biomass and medicinal compound accumulation. Therefore, the superior provenances identified here (e.g., Songyang and Yunhe) warrant further validation in long-term field trials to confirm their biomass yield performance at commercial harvest stages. Such extended evaluations will ultimately determine their true economic value for understory cultivation.” **It has been added in the line 514-523.**

3. Comments: *The trade-off between saponin content and overall performance in SY/YH provenances is well-handled, but the practical implications for breeders could be elaborated slightly more.*

Response: we have revised Section 4.3 to expand the discussion on the trade-off between saponin content and overall performance in the SY and YH provenances:

“This trade-off has important practical implications for breeding programs. For breeders targeting general-purpose cultivars with broad-spectrum medicinal quality and robust growth, SY and YH represent the optimal choice. However, for specialized breeding objectives focused specifically on high saponin production—such as developing cultivars for saponin-based pharmaceutical applications—these Cluster III provenances should be prioritized. Furthermore, future cross-breeding strategies could aim to combine the high polysaccharide/flavonoid traits of SY/YH with the high saponin traits of TG or JJ through controlled hybridization, potentially generating elite lines that integrate multiple desirable characteristics. This targeted approach to provenance selection based on specific breeding goals lays a foundation for the diversified utilization of *P. cyrtonema* germplasm resources, consistent with the findings of Peng et al. [57].” **It has been added in the line 477-488.**

4. Comments: *The map copyright issue is resolved, but ensure the final figure caption matches the statement in the response.*

Response: We have ensured that the final figure caption in the manuscript matches the statement provided in our response. The figure caption for Figure 1 will read:

“Fig 1. Distribution map of provenance collection sites and experimental sites for *P. cyrtonema*. (a) collection sites and experimental sites; (b) *P. cyrtonema* in Jinyun sites; (c) Rhizomes. The map was created by the authors using geographic coordinates and is for illustrative purposes only.”

**Heritability & Replication:**

Comments: *You note that heritability estimates are consistent with other medicinal plant trials. Could you provide a brief comparative reference or range from those studies (e.g., Dendrobium, Astragalus) in the manuscript for context?*

Response: In the revised manuscript, we have included comparisons with **heritability data** estimates from other understory medicinal plants (***Panax ginseng* (0.63–0.96)*, Picrorhiza kurrooa*(0.65-0.86)**) to further corroborate the robustness of our findings, while also acknowledging the limitations of the current study:

“Although environmental effects significantly affected trait expression across different sites in our study, the provenance repeatability remained notably high (0.63-0.99). This pattern shows similarities to observations in other medicinal plants, such as ***Panax ginseng* (0.63–0.96) [42] and *Picrorhiza kurrooa* (0.65-0.86)** [43], indicating substantial heritability for these traits and suggesting significant potential for genetic gain through selection [44].” **It has been revised in the line 359-361.**

We have changed the reference.

[42] Zhao, S.J., Li, F.Y., Zhao, Y.H., et al. Studies on Selection Theory for High-yield Variety of Panax ginseng and Breeding of Jishen 1. *Sci. Agr. Sin.* 1998, 31(5).

[43] Hemdan, A., Chauhan, R.S., Pant, S.C. et al. Variability and heritability for yield and yield attributing characters in an endangered medicinal herb Picrorhiza kurrooa Royle ex Benth under cultivation in Bharsar, Uttarakhand, Western Himalaya, India. *Genet. Resour. Crop. Evol.*2025*,* 72, 8079–8099.

**Breeding Strategy:**

Comments: *Given the trade-off between high saponin content and overall performance in SY/YH, what specific breeding strategy do you recommend for developing cultivars targeting high saponin versus balanced performance? Would you propose a hybridization program between SY/YH and high-saponin provenances like TG?*

Response: we have revised Section 4.3 to expand the discussion on the trade-off between saponin content and overall performance in the SY and YH provenances:

“This trade-off has important practical implications for breeding programs. For breeders targeting general-purpose cultivars with broad-spectrum medicinal quality and robust growth, SY and YH represent the optimal choice. However, for specialized breeding objectives focused specifically on high saponin production—such as developing cultivars for saponin-based pharmaceutical applications—these Cluster III provenances should be prioritized. Furthermore, future cross-breeding strategies could aim to combine the high polysaccharide/flavonoid traits of SY/YH with the high saponin traits of TG or JJ through controlled hybridization, potentially generating elite lines that integrate multiple desirable characteristics. This targeted approach to provenance selection based on specific breeding goals lays a foundation for the diversified utilization of *P. cyrtonema* germplasm resources, consistent with the findings of Peng et al. [57].” **It has been added in the line 477-488.**

**Long-term Yield:**

Comments: *While one-year data is suitable for early selection, how do you anticipate the ranking of provenances might change after 3–5 years of growth, particularly for rhizome biomass? Do you have preliminary multi-year data or plans for follow-up trials?*

Response: Based on our ongoing observations and the broader literature on perennial plant provenance trials, we anticipate that the relative rankings of provenances for key traits will remain largely stable over 3–5 years of growth, for the following reasons:

High heritability of key traits: The provenance repeatability estimates in our study (0.63–0.99, with most growth traits exceeding 0.90) indicate strong genetic control of the measured traits. In perennial species, traits with high heritability typically exhibit stable ranking patterns across developmental stages. Consistency with other perennial medicinal plants: Studies on *Panax ginseng* and *Picrorhiza kurrooa* have demonstrated that early-stage (1–2 year) rankings for both growth and bioactive compound accumulation are generally predictive of mature plant performance, particularly when multi-environment testing is employed as in our study. Observed patterns in our data: The consistent superiority of SY and YH provenances across all three environmentally distinct sites in the first year suggests broad adaptation rather than site-specific chance, which would likely persist over time.

However, we fully acknowledge that absolute values (particularly rhizome biomass) will increase substantially with age, and the magnitude of differences between provenances may change even if relative rankings remain stable. We are therefore actively conducting follow-up observations on the same trial populations.

Regarding follow-up trials:

All three experimental sites have been maintained, and we are currently in the third year of growth monitoring (2022–2025). Annual measurements of growth traits continue, and we plan to conduct comprehensive harvests and medicinal component analyses at Year 5 to validate the stability of provenance rankings and to quantify biomass yield at commercial maturity

Environmental Drivers:

Comments: *You identified clear geo-climatic drivers for saponins and flavonoids. Were soil properties (pH, nutrients) measured or considered? If not, do you think including them in future models would significantly improve predictive accuracy for medicinal component accumulation?*

Response: We acknowledge that soil factors such as pH and nutrient availability can significantly affect secondary metabolite synthesis in medicinal plants, as demonstrated in numerous studies. In our current study, soil properties were not measured, as our primary focus was on the effects of climatic and geographic factors (temperature, precipitation, sunshine duration, latitude, longitude, altitude) across the three heterogeneous garden sites. All sites were managed under uniform cultivation practices to minimize confounding effects from agronomic variables.

However, we fully agree that incorporating soil parameters in future models would likely enhance predictive accuracy. Recent studies have shown that soil properties such as organic matter content, available potassium, and trace elements can significantly modulate the accumulation of bioactive compounds. We also mentioned this point in the Study Limitations section (Section 4.4) of the manuscript.

“Complex field factors (soil types, microbiome communities) were not systematically quantified [38], possibly constraining the generalizability of conclusions.”

We thank you for highlighting this important dimension, which will guide the design of our future multi-factor studies.

Practical Recommendations for Farmers:

Comments: *For a farmer in a high-altitude, dry region of Zhejiang, which provenance would you recommend based on your findings, and why? Could you provide* *a simplified decision matrix or flowchart for growers in different eco-zones?*

Response: Based on the geo-climatic drivers identified in our study and the performance patterns observed across provenances, we can provide the following guidance.

For a farmer in a high-altitude, dry region of Zhejiang, we would recommend the Yunhe (YH) provenance as the primary choice, with the Songyang (SY) provenance as a strong alternative.

The rationale is as follows:

Flavonoid accumulation: Our correlation analysis (Fig. 2) showed that flavonoid content is highly significantly positively correlated with longitude and significantly negatively correlated with annual rainfall. This indicates that drier conditions promote flavonoid synthesis. The YH provenance consistently exhibited the highest flavonoid content across all three trial sites (2.78–3.48 mg·g⁻¹), making it ideally suited for drier environments.

Adaptation to altitude: Both YH and SY originate from mid-elevation areas (534–645 m) in southern Zhejiang and demonstrated superior performance across all sites, including the high-altitude Jinyun site. Their broad adaptation suggests they can thrive under the environmental stresses associated with higher elevations.

Balanced medicinal quality: While saponin content is positively correlated with altitude and temperature, the YH provenance maintains respectable saponin levels (21.29–24.78 mg·g⁻¹) while excelling in other medicinal components. For a grower seeking reliable overall quality rather than extreme values in a single compound, YH offers the best balance.

**In response to your suggestion, we have designed a simplified decision matrix for growers in different eco-zones (Table 8).**

**We believe this provides growers with clear, science-based guidance while acknowledging the need for site-specific validation. Thank you for encouraging us to make our findings more accessible and actionable for the farming community.**

Table 8 Simplified decision matrix for growers in different eco-zones

| Eco-zone Characteristics | Recommended Provenance(s) | Primary Strengths | Considerations |
| --- | --- | --- | --- |
| High-altitude, dry regions | YH (Yunhe) | Highest flavonoid content, good saponin accumulation, broad adaptation | Moderate polysaccharides |
| High-altitude, high-rainfall regions | SY (Songyang) | Highest polysaccharides and total phenolics, excellent growth vigor | Moderate flavonoids |
| Low-altitude, warm regions | JS (Jiangshan) | High saponin content, good growth performance | Variable across sites |
| Saponin-focused production | TG (Tonggu) or JJ (Jiujiang) | Exceptionally high saponin | Lower in other medicinal components |
| General-purpose cultivation | SY or YH | Balanced medicinal profile; superior growth, stable across environments | Best overall choice for most growers |

Data Availability:

Comments: *You state all data are in the manuscript. Are the raw datasets (e.g., trait measurements per plant) available as supplementary files or in a repository? This would enhance reproducibility.*

Response: we have prepared the raw measurement data for all individual plants across the 11 provenances and three experimental sites as a supplementary file. This dataset includes: (1) growth trait measurements (plant height, stem diameter, leaf length, leaf width, leaf area, leaf perimeter) for each sampled plant; (2) medicinal component concentrations (polysaccharides, saponins, flavonoids, total phenolics) per individual rhizome sample; and (3) provenance and site identifiers to allow full replication of our analyses. The data are provided in Excel format (.xlsx) as **Supplementary File S1 and S2**. We have also updated the Data Availability Statement in the manuscript accordingly to indicate that all raw data supporting the findings are available in the supplementary material. We believe this addition will significantly strengthen the reproducibility and utility of our work for the research community.

Limitations and Future Work:

Comments: *You mention future work could use AMMI or GGE biplots. Do you plan to re-analyze this dataset with these methods, or are you referring to future trials? If the former, could including such an analysis in the supplementary materials strengthen the current paper?*

Response: After careful consideration, we have decided not to re-analyze the current dataset with these methods, as our primary objective was to conduct an initial comparative screening of provenances—a goal effectively addressed through our combined ANOVA, PCA, and cluster analysis. These methods have successfully quantified variation, identified key environmental drivers, and classified provenances into performance-based groups. AMMI and GGE biplots, while powerful tools for visualizing genotype-by-environment interactions, are most informative when applied to multi-year datasets that capture temporal stability patterns; our single-season data, though valuable for preliminary selection, may not fully support the robust stability assessments these methods are designed to provide. Therefore, our reference to these approaches in the manuscript pertains specifically to future multi-year trials, which we are currently conducting across all three experimental sites. Once we accumulate data from multiple harvest years (e.g., Year 3 and Year 5), we plan to apply stability analysis methods—including AMMI or GGE biplots—to validate ranking stability and identify provenances with broad versus specific adaptability across both sites and growing seasons. We have clarified this distinction in the revised Section 4.4 to ensure readers understand that these methods are intended for subsequent phases of our ongoing research program. Thank you again for this valuable suggestion, which has helped us articulate a clearer path forward.

We believe these revisions have further strengthened the manuscript and clarified its contributions. We hope it is now acceptable for publication. If you have any further queries, please do not hesitate to contact us.

Sincerely yours

Xiaodeng Shi

Mar. 19, 2026

*Zhejiang Academy of Forestry, Hangzhou, China.*
